# Internet-related problems and learning motivation of the students of medical university

**DOI:** 10.1192/j.eurpsy.2021.2003

**Published:** 2021-08-13

**Authors:** E. Sedova, T. Goryacheva, M. Zavadskaya

**Affiliations:** Psychological-social Department, Pirogov Russian National Research Medical University, Moscow, Russian Federation

**Keywords:** Internet addiction, motivational sphere

## Abstract

**Introduction:**

Nowadays the cyberspace penetrates all the spheres of our lives: work, leisure and learning activity. However, uncontrolled presence in the virtual reality can form Internet-addictive behavior. Young people seem to be in increased risk of Internet-related problems.

**Objectives:**

The research aim is to study the motivational shpere of the students with different level of Internet dependency.

**Methods:**

The research methods are: Chen Internet Addiction Scale (CIAS), Internet Perception Inventory, Learning Motivation Diagnostics Inventory, Test of Motivation of Success or Fear of the Failure. The sample consists of 37 students of the medical university in the age from 21 to 24 years. According to the results of the CIAS 3 groups have been marked out: Group 1 - with the highest level of Internet-related problems, Group 2 – the risk group of forming the Internet addiction, Group 3 – students who have not demonstrated proneness to Internet-addictive behavior.

**Results:**

The motivational sphere of the students with a low risk of Internet addiction seem to be more differenciated comparing with the one of the rest students. The motives of creative self-realization; communicative, social and learning motives have been demostrated. However, we have not found a significant differance between the groups in motivation for success. The motives of professional self-realization are equally important for all the research participants.

**Conclusions:**

The obtained data can be implemented when designing Internet addiction prevention programs. We assume that including the motivational component into such programs can make them more effective.

**Disclosure:**

No significant relationships.

